# Stunting and anemia among children 6–23 months old in Damot Sore district, Southern Ethiopia

**DOI:** 10.1186/s40795-018-0268-1

**Published:** 2019-01-07

**Authors:** Bereket Geze Malako, Benedict Oppong Asamoah, Minyahil Tadesse, Robel Hussen, Meklit Tesfaye Gebre

**Affiliations:** 1 World Vision Ethiopia, Jimma area cluster office, Gewata area development program, Jimma, Ethiopia; 2 Social Medicine and Global Health, Department of Clinical Sciences, Malmo, Lund University, Lund, Sweden; 3 Health Sciences and Medicine College, Wolaita Sodo University, Wolaita Sodo, Ethiopia; 4 School of public health, Dilla University, Dilla, Ethiopia; 5 School of Public Health and Medical Sciences, Jimma University, Jimma, Ethiopia

**Keywords:** Stunting, Anemia, Southern Ethiopia

## Abstract

**Background:**

Stunting and anemia are long-standing public health challenges which adversely affects the cognitive development and physical wellbeing of children in low income settings. The aim of this study was to assess the prevalence and associated factors of stunting and anemia among 6–23 months old children in Damot Sore District, Southern Ethiopia.

**Methods:**

Cross-sectional survey was conducted among 477 children aged 6–23 months, which were living in Damot Sore District, in April 2017. A multistage sampling technique was used. Villages were randomly selected and systematic random sampling method was used to select study participants. Data on socio-demographic, anthropometric, dietary, blood samples for hemoglobin were collected. Data were entered into EPI Data V. 3.1 and exported into SPSS Version 21.0 for analysis. A principal component analysis (PCA) was done to generate wealth score of households. Binary logistic regression model was used to identify factors associated with the outcome variables (stunting and anemia) separately, those variables having less than a *p*-value of 0.25 were chosen as candidate for multivariable analyses and finally multivariable logistic regression model was used to identify independent variables of each outcomes, with statistical significance set at *p* < 0.05 (95% confidence interval (CI)).

**Results:**

Out of 477 children studied, 31.7% were stunted and 52% were anemic. In the multivariable analyses, the number of under five children within a household (AOR = 4.18, 95% CI: 2.65–6.57), drinking water from unsafe source (AOR = 4.08, 95% CI: 1.33–12.54) and anemia (AOR = 3.13, 95% CI 2.00–4.92) were factors significantly associated with stunting. On the other hand, independent variables of anemia were early initiation of complementary feeding (AOR = 2.96, 95% CI: 1.23–4.85), poor dietary diversity (AOR = 2.95, 95% CI: 1.78–4.91), poor breast feeding practice (AOR = 2.94, 95% CI: 1.63–5.32) and stunting (AOR = 3.65, 95% CI: 2.15–6.19).

**Conclusion:**

This study revealed higher level of stunting and anemia among children aged 6–23 months than WHO (world health organization) criteria of public health importance. Sustainable promotion of diversified diet, optimal complementary feeding, optimal and complementary breast feeding practices, improving sanitation infrastructure are measures needed to tackle these severe public health challenges.

## Background

Significances of early life experiences to subsequent health outcomes are main public health concerns and increasingly gaining attention in the scientific literature [1]. Early nutritional malignancies such as stunting and anemia have been suggested to cause irreversible health effects of later life courses such as acute and chronic diseases, non-communicable diseases, cognitive development and economic productivity of individuals and the society [1, 2].

Anemia (Hb < 110 g/L), which could originate from low consumption or absorption of the diet or blood loss and inability to absorb nutrients as a result of infection from intestinal worms is believed to be highly prevalent during infancy. One fourth of the global population is affected by anemia and about 42.6% of them are children [2]. Various surveys carried out in resource limited settings revealed that the incidence of stunting is at its highest point in children of the age group from 6 to 23 months, because within this period children have a greater demand for macro and micronutrients which are vital for child growth. However, inadequate access to and affordability of quality and sufficient complementary foods, frequent disease episodes, poor sanitation and hygiene practice have been identified as the main causes of malnutrition [2–5].

Anemia might occur at any time and at all stages of the lifespan, but children are the most at risk segment of the population in developing countries, and sub-Saharan Africa bears the highest burden of anemia [3].

Children aged 6 to 23 months have high iron requirements as they are believed to show extremely rapid growth pattern during this period. Trends in Ethiopia indicates that, the magnitude of anemia among 6–23 months old children increased over 10% between 2011 and 2016 [4, 5].

According to the Ethiopian Demographic Health Survey (EDHS) key indicators report of 2016, 38% of children below five years of age were stunted nationally, and 38.6% of under-five children were stunted also in Southern Nations Nationalities and Peoples Region (SNNPR); this shows that there is chronic severity and burden of malnutrition in the country’s every corner [5]. A study conducted in Kemba Woreda of Southern Ethiopia also revealed that 18.7% of children were stunted [6]. Comparisons of two agro-ecological areas in northern and eastern part of Ethiopia indicated that 36.2 and 42.6% of children aged 6–23 months old were stunted, respectively [7].

Therefore, this study determined the prevalence and predictors of stunting and anemia among children aged 6–23 months old and associated factors in Damot Sore District, Southern Ethiopia.

## Methods

### Study area and setting

Community based cross-sectional survey was carried out among 6–23 months old children residing in 6 villages of Damot Sore District, situated in Southern Nation Nationalities and Peoples Region (SNNPR), which is 326 km from the capital city of Ethiopia. All children in the aforementioned age group who stayed in the study area during the time of the interview were qualified to take part in the study.

### Sample size and sampling procedure

A formula for estimation of single population proportion was used to calculate the sample size. The following criterions were applied to estimation of the sample size; 95% confidence level, 5% error of margin, and 66.6% prevalence of anemia among children aged 6–23 months old – taken from a previous study in a rural area in northern Ethiopia [8]. The estimated sample size was then adjusted for a non-response rate of 5% and multiplied by the design effect of 1.5 to obtain a final estimate of 498, the sample size used for this study.

Study participants were selected by a multi – stage sampling technique. From a total of 20 rural villages, 6 were randomly selected using a lottery method.

Sampled number of children per village were proportionally allocated based on their number of households. Finally, using family folder which is found in community health information system (CHIS) of village health posts as a sampling frame, households with children 6–23 months old were selected by simple random sampling method. For a household that have twins or more than one child resident there, one of them was selected by using lottery method. In case of absence of an eligible child in a given household, a substitution was made by a child in the next household. Children with physical deformities of limbs, spine, suffering from diseases and have mental defects were excluded. In addition to this, children who had received blood transfusion and anti helmenthiasis prior (two months) to data collection were excluded.

### Data collection

Data were collected using the questionnaire adopted from previous studies [4, 9] and pre-tested before the start of this study. The questionnaire was first prepared in English language and then translated to Amharic language. Prior to collection of data, the purpose of this study was explained to the study units; their consent to participate was sought and was also informed that their participation in the study was totally voluntary.

The response from the mother/caregiver was recorded after the data collectors read out the questions loud. Date of interview and date of birth was used to calculate age of the child, because the year of birth is inaccurately announced oftentimes. The nutritional status for all children aged 6–23 months old was assessed by taking Anthropometric measurements.

### Data collectors and measurements

Anthropometric data for this study was collected by six skilled and trained data collectors who administered the questionnaires. Two supervisors closely supervised the process of data collection. Nutritional status was assessed by taking anthropometric body measurements of the children. Length of a child was measured in a recumbent position to the nearest 0.1 cm by using a portable board provided by UNICEF (United Nations Children’s Fund) with an upright movable wooden base. Anthropometric measurements were converted to z-scores of indices using WHO Anthro software [10].

### Laboratory investigations

Hemoglobin count and malaria status of children were investigated. Hemoglobin was measured from capillary blood by aseptically collecting blood sample from the middle finger of study participants, then the analysis have been done by using Automated HemoCue analyser (HEMOCUE Hb 301, HEMOCUE AB, ANGELHOLM SWEDEN) machines and the results were immediately recorded in the field in terms of g/dl. After adjusting the hemoglobin concentration for changes in the altitude and smoking individual within a household, the results were categorized based on the WHO cut off point, which categorizes a child as anemic if the hemoglobin count is less than 11.0 g/dl [11]. Malaria test was done using rapid diagnostic test (RDT) kit, which was commonly used to assess the status of malaria in the community [12]. Blood test for malaria was collected by finger puncture and the result was recorded as positive or negative with regards to species specification.

### Data quality control

Three day training was given for data collectors about study objective, interview techniques, anthropometric measurements and ethical issues during data collection. Rapid diagnostic malaria test results were compared with blood film result by microscope. Standard operating procedures and manufacturer’s instructions were strictly followed starting from sample collection up to result reporting for laboratory activities.

The questionnaire was pre-tested on similar setting outside the study area before the collection of actual data. The principal investigator carefully monitored the data collection process.

Quality of the measurements were ensured by maintaining consistency of anthropometric measurement, data collectors were tested using ENA for SMART software before starting data collection.

*Standardization*: all children were measured without any shoes and clothes were taken off.

Multicollinearity for independent predictors of stunting and anemia were checked and Crombach’s alpha was checked for household wealth. Data cleaning were done and outliers were identified and managed properly before the analysis.

### Data management

The data management were done by using three statistical softwares. During the data collection, completeness and uniformity of the data were checked daily before entry.

The data were first entered into EpiData V.3.1 statistical software for coding. Afterwards the data were transported into the software WHO Anthro, where length-for-age Z-scores were computed and further checks done to ensure that flags resulting from wrongly entered data were corrected. After the initial cleaning, all the z-score values which remained as irregular were cleaned from the file and excluded from further analyses. The cleaned file was then exported to SPSS version 21.0 for further analyses.

### Statistical analyses

Bivariate and multivariable logistic regression was used to examine the association between stunting, anemia and the explanatory variables. From the binary regression models, independent variables which were associated with the outcome at *p*-value less than 0.25 were selected as candidate for inclusion in the multivariable logistic regression models. Statistical significance was set at *p* < 0.05 and 95% confidence interval.

### Operational definition

**Stunting**: is defined as length-for-age Z-scores below minus two <− 2 Z score or Standard deviation of the reference population of World Health Organization (WHO) Multicentre Growth Study. Severe stunting is defined as LAZ scores below minus three <− 3 Z score or Standard deviation of the reference population of WHO Multicentre Growth Study [10].

**Anemia**: A child is considered to be anemic if the hemoglobin count is less than 11.0 g/dl against the WHO reference range [11].

**Poor DDS**: dietary diversity of less than 4 food categories.

**Good DDS**: dietary diversity of more than or equal to 4 food categories.

**Poor breast feeding practice**: failed to breast for at least 8 times per day or inappropriate baby position or switching to the next breast without finishing.

**Good breast feeding practice**: breast feed for more than or equal to 8 times a day or appropriate baby position or switching to the next breast after finishing one.

## Results

### Socio-demographic characteristics of children and mothers

From a total of 498 children participated in the study, 477 children were involved in the study yielding a response rate of 95.78%. Twenty-one 21(4.22%) of sampled children were dropped from the analysis due to the incompleteness of outcome variables. Mean age of children and mothers were 13.69 (±5.41) months and 30.11 (±5.78) years, respectively. As shown in Table 1 among the total households surveyed, 258 (54.1%) has a total family size greater than five while half of them has more than one under five children. Two hundred and twenty-three (46.8%) mothers introduce complementary feeding in any other months than just at six months (Table).

**Table 1:** Child and parents related characteristics among children aged 6–23 months in Damot Sore District, Southern Ethiopia, from March to April 2017

Characteristics	Categories	Frequency (*N* = 485)	Percent (%)
Sex of the child	Male	243	50.9
	Female	234	49.1
Age of the mother	15–24 years	84	17.6
	25–34 years	246	55.3
	35–49 years	129	27.0
Age of the child	6–11 months	190	39.8
	12–17 months	160	33.5
	18–23 months	127	26.6
Educational status of mother	No formal education	307	64.4
	Formal education	170	35.6
Educational status of father	No formal education	238	49.9
	Formal education	236	49.5
Mother’s occupation	Unemployed	459	96.2
	Government/private employee	18	3.8
Father’s occupation	Unemployed	454	95.2
	Government/private employee	19	4
Total number of family size within households	Less than or equal to 5	219	45.9
	Greater than 5	258	54.1
Number of under five children within household	More than one child	240	50.3
	One child	237	49.7
Wealth	Low	201	42.1
	Middle	72	15.1
	High	204	42.8
Introduction time of complementary feeding	Earlier than 6 months	223	46.8
	Just at 6 months	254	53.2
Breast feeding practice	Poor	113	23.7
	Good	331	69.4
	Never breast feed at all	33	6.9
Source of drinking water	Piped inside compound	36	7.5
	Public	351	73.6
	Protected well/spring	90	18.9
Toilet	No facility/bush/field	12	2.5
	Have latrine	465	97.5
Utilization of Insecticide Treated Net (ITN)	Not appropriately	45	9.4
	Appropriately	404	84.7
	Never had ITN at all	28	5.9
Having diarrhoea	No	313	65.8
	Yes	163	34.2
Having Malaria	Yes	21	4.4
	No	456	95.6
Having low dietary diversity score	Yes	313	65.6
	No	164	34.4

#### Prevalence of stunting among children

**Fig. 1:**
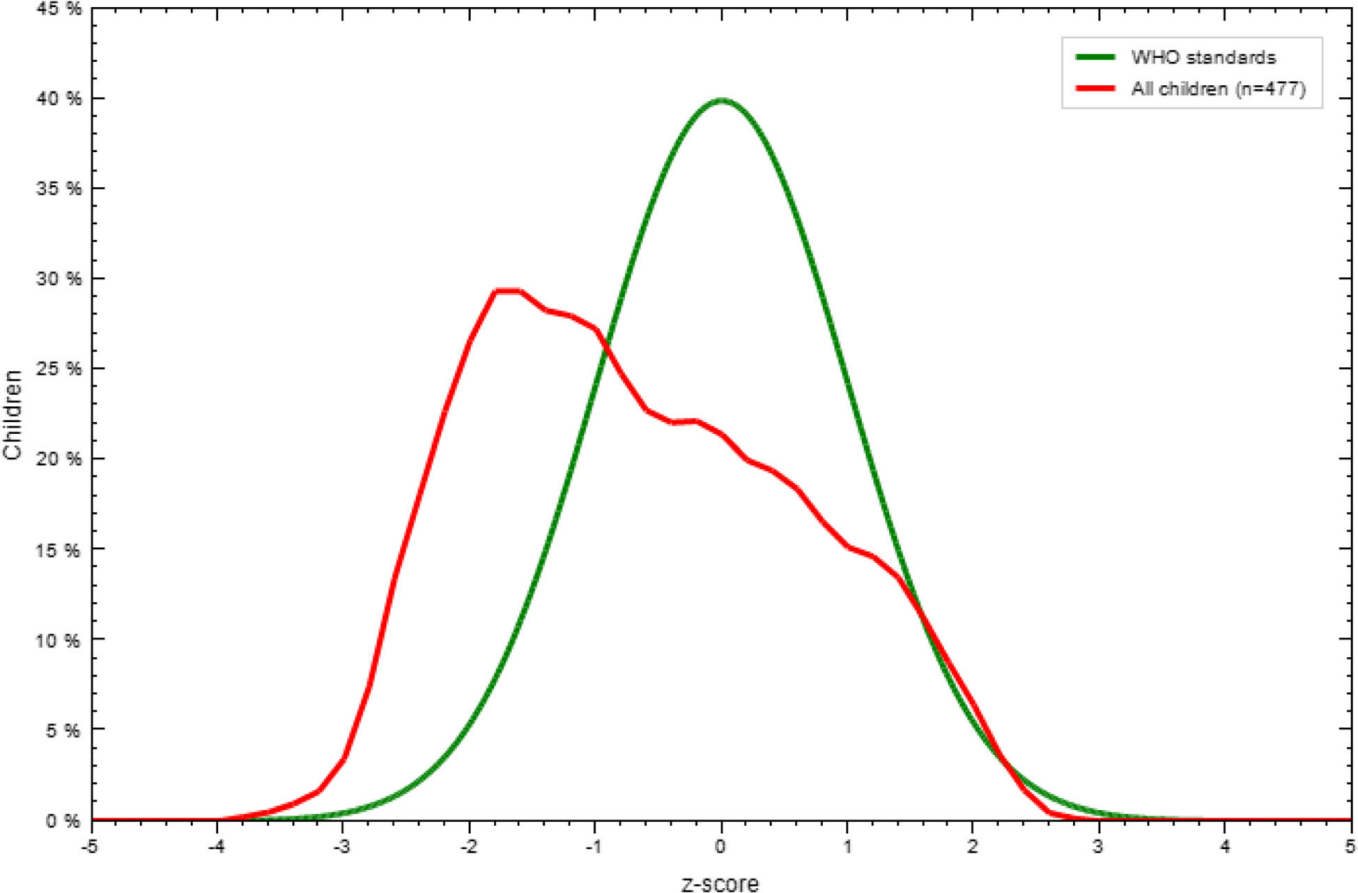
LAZ-scores compared to WHO growth standards in Damot Sore district, Southern Ethiopia, from March to April 2017

The burden of stunting among the study units was 31.7% and the mean (±SD) stunting (LAZ) of the children was − 0.92 (±1.31). As demonstrated in Fig. 1, Z-score curves were shifted to left of the WHO growth reference curve, which shows stunting, is prevalent in the study area (Fig. 1).

In bivariate analysis as shown in Table 2, age of mothers, age of the children, mothers occupation, number of under five children within household, wealth, source of drinking water, toilet, dietary diversity score and anemia were associated with stunting (Table 2).

**Table 2:** Bivariate analysis that shows independent variables of stunting among children in Damot Sore District, Southern Ethiopia, from March to April 2017

		Stunting status		COR (95% C.I.)	*P*
		Stunted	Not stunted		
Characteristics	Categories				
Sex of the children	Male	72	171	0.83(0.56–1.22)	0.33
	Female	79	155	1	
Age of mothers	15–24 years	35	49	1.41(0.86–2.33)	0.17^*^
	25-34 years	70	194	1.49(0.89–2.93)	0.13^*^
	35-49 years	46	83	1	
Age of the children	6–11 months	63	127	1.29(0.73–2.26)	0.38
	12–17 months	55	105	0.65(0.41–1.02)	0.06^*^
	18-23 months	33	94	1	
Educational status of mothers	No formal education	94	213	0.87(0..59–1.31)	0.51
	Formal education	57	113	1	
Educational status of fathers	No formal education	78	160	1.13(0.77–1.67)	0.53
	Formal education	71	165	1	
Mothers occupation	Unemployed	149	310	3.84(0.87–16.94)	0.07^*^
	Government/private employee	2	16	1	
Fathers occupation	Unemployed	145	309	1.76(0.57–5.39)	0.32
	Government/private employee	4	15	1	
Total number of family size within households	Greater than 5	86	172	1.18(0.80–1.74)	0.39
	Less than or equal to 5	65	154	1	
Number of under five children within household	More than one child	111	129	4.24(2.77–6.477)	0.001^*^
	One child	40	197	1	
Wealth	Low	66	135	1.23(0.80–1.88)	0.34
	Middle	27	45	1.51(086–2.66)	0.15^*^
	High	58	146	1	
Introduction time of complementary feeding	Earlier than 6 months	69	154	0.94(0.64–1.38)	0.75
	Just at 6 months	82	172	1	
Breast feeding practice of mothers	No	38	75	1.14(0.72–1.79)	0.58
	Yes	102	229	1	
Source of drinking water	Public and other sources	147	292	4.28(1.49–12.29)	0.04^*^
	Piped inside compound	4	34	1	
Toilet	No facility/bush/field	1	11	0.19(0.02–1.49)	0.11^*^
	Have latrine	150	315	1	
Having Diarrhoea	Yes	50	113	1.04(0.57–1.88)	0.89
	No	23	50	1	
Having Malaria	Yes	7	14	1.08(0.43–2.74)	0.87
	No	144	312	1	
Dietary diversity score	Poor	110	203	1.63(1.06–2.48)	0.02^*^
	Good	41	123	1	
Growth monitoring and promotion service utilization	No	144	304	1.49(0.62–3.56)	0.37
	Yes	7	22	1	
Anemia	Anemic	45	184	3.05(2.02–4.61)	0.001^*^
	Not anemic	106	326	1	

*variables with *p* < 0.25

#### Prevalence of anemia

This study revealed that the magnitude of anemia was found to be 52% after adjusting for altitude and smoking individual within a household based on WHO Hb adjusting measurements. In bivariate analysis as shown in Table 3, age of mothers, age of the children, mothers educational status, fathers occupation, number of under five children within household, introduction time of complementary feeding, breast feeding practice of mothers, source of drinking water, toilet, dietary diversity score, growth monitoring and promotion service utilization and stunting were associated with anemia (Table 3).

**Table 3:** Bivariate analysis that shows independent variables of anemia among children in Damot Sore district, Southern Ethiopia, from March to April 2017

Variables		Anemia status		COR (95% C.I.)	*p*
		Anemic	Not anemic		
Characteristics	Categories				
Sex of the children	Male	124	119	0.92(0.64–1.32)	0.67
	Female	124	110	1	
Age of the mother	15–24 years	44	40	1.30(0.75–2.26)	0.34
	25–34 years	145	119	1.44(0.95–2.20)	0.09^*^
	35-49 years	59	70	1	
Age of the child	6–11 months	105	85	1.72((1.09–2.72)	0.19^*^
	12-17 months	90	70	1.79(1.12–2.87)	0.02^*^
	18-23 months	53	74	1	
Educational status of mother	No formal education	166	141	1.26(0.87–1.84)	0.22^*^
	Formal education	82	88	1	
Educational status of father	No formal education	126	112	1.09(0.75–1.56)	0.65
	Formal education	120	116	1	
Mother’s occupation	Unemployed	241	218	1.74(0.66–4.56)	0.26
	Government/private employee	7	11	1	
Father’s occupation	Unemployed	243	211	6.14(1.76–21.37)	0.01^*^
	Government/private employee	3	16	1	
Total number of family size within household	Greater than 5	134	124	0.99(0.69–1.43)	0.98
	Less than or equal to 5	114	105	1	
Number of under five children within household	More than one child	137	103	1.51(1.05–2.17)	0.03^*^
	One child	111	126	1	
Wealth	Low	103	98	0.99(0.67–1.46)	0.96
	Middle	40	32	1.18(0.68–2.02)	0.55
	High	105	99	1	
Introduction time of complementary feeding	Earlier than 6 months	142	81	2.45(1.69–3.54)	0.001^*^
	Just at 6 months	106	148	1	
Breast feeding practice of mother	No	80	33	2.99(1.89–4.74)	0.001^*^
	Yes	148	183	1	
Source of drinking water	Unprotected well	179	172	1.84(0.90–3.75)	0.09^*^
	Protected well/spring	56	34	2.91(1.31–6.50)	0.01^*^
	Piped inside compound	13	23	1	
Toilet	No facility/bush/field	9	3	2.84(0.76–10.61)	0.12^*^
	Have latrine	239	226	1	
Utilization of insecticide treated bed net (ITN)	No	24	21	1.08(0.58–1.99)	0.81
	Yes	208	196	1	
Having diarrhoea	Yes	97	66	0.97(0.55–1.70)	0.91
	No	44	29	1	
Having malaria	Yes	10	11	0.83(0.34–1.99)	0.68
	No	238	218	1	
Dietary diversity score	Poor	190	123	2.82(1.90–4.18)	0.001^*^
	Good	58	106	1	
Growth monitoring and promotion service utilization	No	239	209	2.54(1.13–5.70)	0.02^*^
	Yes	9	20	1	
Stunting	Stunted(<-2SD)	106	45	3.05(2.02–4.60)	0.001^*^
	Not stunted (≥-2SD)	142	184	1	

*variables with *p* < 0.25

#### Independent variables associated with stunting after adjusting for other variables

Source of drinking water, status of anemia, age of the child, age of the mother, wealth, availability of toilet, number of children under five years of age living in a household, occupation of mother, were found to be statistically significant in bivariate analysis. In a multivariable logistic regression analysis which was as shown on Table 4, the number of under five children within household (AOR = 4.18, 95% CI: 2.65–6.57), drinking water from unsafe source (AOR = 4.08, 95% CI: 1.3312.54) and anemia (AOR = 3.13, 95% CI 2.00–4.92) were factors significantly associated with stunting (Table 4).

**Table 4:** Multivariable logistic regression analysis of factors which have statically significant association with stunting, in Damot Sore district, Southern Ethiopia, from March to April 2017

Associated factors		Stunting status		COR (95% C.I.)	AOR (95% C.I.)
		Stunted	Not stunted		
Number of under five children within household	More than one child	111	129	4.18(2.67–6.57)	4.18(2.65–6.57)^*^
	One child	40	197	1	1
Source of drinking water	Public and other sources	147	292	4.28(1.49–12.29)	4.08(1.33–12.54)
	Piped inside compound	4	34	1	1
Anemia	Anemic	45	184	2.9(2.02–4.61)	3.13(2.00–4.92)^*^
	Not anemic	106	326	1	1

^*^p < 0.001 ^**^Crude odds ratio with 95% confidence interval ^***^Adjusted odds ratio with 95% confidence interval

#### Independent variables associated with anemia after adjusting for other variables

As demonstrated in Table 5, early initiation of complementary feeding (AOR = 2.96, 95% CI: 1.23–4.85), poor dietary diversity (AOR = 2.95, 95% CI: 1.78–4.91), poor breast feeding practice (AOR = 2.94, 95% CI: 1.63–5.32) and stunting (AOR = 3.65, 95% CI: 2.15–6.19) were factors associated with anemia in multivariable logistic regression (Table 5).

**Table 5:** Multivariable logistic regression analysis of factors which have statically significant association with anemia, in Damot Sore district, Southern Ethiopia, 2017

Associated factors		Anemia status		COR** (95% C.I.)	AOR*** (95% C.I.)
		Anemic	Not anemic		
Dietary diversity score	low	190	123	2.82(1.90–4.18)^*^	2.95(1.78-4.91)^*^
	high	58	106	1	
Introduction time of complementary feeding	Earlier than 6 months of age	142	81	2.45(1.69–3.54)	2.96(4.85)
	Just at 6 months of age	106	148	1	
Breast feeding	No	80	33	2.99(1.89–4.74)	2.94(1.63–5.32)
	Yes	148	183	1	
LAZ	<2SD	106	45	3.05(2.02–4.60)	3.65(2.15–6.19)
	Normal	142	184	1	

^*^p < 0.001 ^**^Crude odds ratio with 95% confidence interval ^***^Adjusted odds ratio with 95% confidence interval

## Discussion

This study indicated that out of 477 sampled children aged 6–23 months old, 31.7% were stunted and 52% were anemic, which could be described as severe public health challenge according to the WHO criteria [13]. This study investigated that, the magnitude of stunting in our study is nearly same as a study conducted in Shey Bench District, southwest Ethiopia (33.3%) [14], but much lower than studies conducted in Dabat District (58.1%) [15] and East Belesa District (57.7%) [16] north west Ethiopia respectively and Hosanna town, southern Ethiopia (35.4%) [17] and EDHS-2016 report for SNNPR among children 6–59 months was 38.6% [5]. However, the result of this study was much higher than a study conducted in Kemba District southern Ethiopia in which 18.7% children were stunted [6]. This might be due to inappropriate infant and young children feeding practice such as non-diversified diet and inconsistent breast feeding.

Children who drink water from unprotected well have higher risk of being stunted than their counterparts who drink tap water. This is accordant with study conducted in different parts of Ethiopia [18–20]. This might occur as a result of utilization of unimproved drinking water sources and poor sanitation which are directly linked with chronic childhood growth retardation.

This study shows that children living in households having more than one under-five aged children was more stunted as opposed to households with a child less than 5 years of age. This in agreement with a study conducted in Eastern Ethiopia [21], Ethiopian Somali region [22], Mozambique [23], Kenya [24] and Ghana [25]. Under-five children living in households with many siblings of same age category in a low-income setting were subjected to increased competition for resources which results in major child health constraints such as stunting and nutritional deficiencies [26].

According to this study, the prevalence of anemia is higher than the EDHS 2016 report of SNNPR under 5 years of age [27] and much lower than studies conducted in northern (66.6%) [8] and eastern (53.7%) [7] parts of Ethiopia, Cameron (66.7%), Sudan (86%) and Uganda (58.8%) [28–30] respectively. This might be attributed to seasonal food shortage since data were collected in spring which is a sunny season characterized by poor consumption of diversified foods and also due to the change made by the existing public health interventions, provision of health information through health extension workers.

Children exposed to lower dietary diversity were 2.95 times more anemic as compared to their counterparts exposed to a higher dietary diversity. This is in line with a study conducted in Wag-Himra, northern Ethiopia, in which poor micronutrient bioavailability related with anemia was observed [31]. This could be due to seasonal unavailability of citric fruits which enhances iron absorption and the socio-economic barriers to provide animal source foods such as meat.

The finding of this study observed that, children who started complementary feeding earlier than 6 months were 2.96 times more likely to develop anemia than children who start at 6 months. On the contrary, a study conducted in Nepal [32] and a systematic review [33] revealed that early introduction of complementary foods had improved hemoglobin concentrations of children. This study is consistent with a study conducted in northern Ethiopia [8], Lebanon [34], Brazil [35] and China [36]. Which reports that early introduction of solid or liquid foods is related with childhood anemia. Early exposure of infants before 6 months of age increases the risks of infections and mal-absorption. This might be due to lack of knowledge about adequacy of excusive breast feeding alone to infants; and thus, they introduce at least cow milk earlier than 6 months.

Government of Ethiopia engaged in many actions to tackle nutritional problems, among them social protections, national nutrition program, community based nutrition, micronutrient supplementations, Seqota declarations and other strategies were used but the problems are still at their climax [4, 5].

Ghana has a history of implementing integrated anemia control programs and reduced prevalence of anemia though multi-sectoral collaboration, home fortification of foods with multiple micronutrient powders for children 6–23 months, simultaneously with malaria prevention [37]. Similarly, to reduce stunting ‘Seqota’ Declaration is a special commitment of government of Ethiopia which will be achieved under the NNP, will be implemented by multi-sectors and on a progress [38]. Implementation of this study is, to update the level of stunting and anemia in this area which will help respecting government offices (Ministry of health, agriculture, education, etc.), as an input for stimulating its efforts to achieve its plan of improving the productivity of individuals and national GDP.

It will also uses as an input to for Growth and Transformation Plan GTP-2 (2016–2020), which were targeted to reduce nutritional problems, through the way it will contribute to the achievement of sustainable development goals.

### Anemia and stunting linkage

This study suggested that childhood growth retardation strongly correlates with anemia in children less than 5 years of age.

This relationship could be ascribed significantly to anemia and stunting as one of the major outcomes of chronic nutritional deficiencies and hemoglobin concentration is used for measurement of child growth and long term deficiency led synergic effect [1, 7].

### Limitation of the study

We encounter a number of limitations: because it is a cross-sectional study, casual inference cannot be made and also it did not show which preceded, whether outcomes or associated factors. Parasite investigation was not done.

## Conclusion

This study revealed higher level of stunting and anemia among children aged 6–23 months than WHO (world health organization) criteria of public health importance. Sustainable promotion of diversified diet, optimal complementary feeding, optimal and complementary breast feeding practices, improving sanitation infrastructure are measures needed to tackle these severe public health challenges. Households with more than one under-five children and unsafe source of drinking water and anemia were factors significantly associated with stunting. On the other hand, early initiation of complementary feeding, poor dietary diversity, poor breast feeding practice and stunting were significantly associated with anemia.

Behavioral change communication on sustainable promotion of diversified diet, optimal and complementary breast feeding practices and sanitation infrastructure are measures needed to tackle these severe public health challenges. Strengthening integration of nutrition intervention activities between and within the existing health facilities and the community on maternal, newborn and child health services. Multiple micronutrient powders (sprinkles) should be initiated by partners and longitudinal studies need to be conducted to identify specific etiologies and root causes of stunting and anemia.

## Abbreviations

ANC: Antenatal Care
AOR: Adjusted Odds Ratio
CBC: Complete Blood Count
CHIS: Community Health Information System
CI: Confidence Interval
COR: Crude Odds Ratio
DDS: Dietary Diversity Score
EDHS: Ethiopian Demographic Survey
FFQ: Food Frequency Questionnaire
GDP: Gross domestic production
GPS: Global Positioning System
GTP: Growth and transformation plan
Hb: Hemoglobin
ITN: Insecticide Treated Bed Net
LAZ: Length-for-Age
OR: Odds Ratio
PCA: Principal Component Analysis
RDT: Rapid Diagnostic Test
SNNPR: Southern Nations Nationalities and Peoples Regional State
UNICEF: United Nations Children’s Fund
WHO: World Health Organization
